# Exploring a novel seven-gene marker and mitochondrial gene TMEM38A for predicting cervical cancer radiotherapy sensitivity using machine learning algorithms

**DOI:** 10.3389/fendo.2023.1302074

**Published:** 2024-01-24

**Authors:** Jiajia Wang, Xue Mou, Haishan Lu, Hai Jiang, Yuejuan Xian, Xilin Wei, Ziqiang Huang, Senlin Tang, Hongsong Cen, Mingyou Dong, Yuexiu Liang, Guiling Shi

**Affiliations:** ^1^ Department of Obstetrics and Gynecology, The Affiliated Hospital of Youjiang Medical University for Nationalities, Baise, China; ^2^ Department of Oncology, The Affiliated Hospital of Youjiang Medical University for Nationalities, Baise, China; ^3^ Clinical Pathological Diagnosis & Research Centra, The Affiliated Hospital of Youjiang Medical University for Nationalities, Baise, China; ^4^ School of Laboratory Medicine, Youjiang Medical University for Nationalities, Baise, China

**Keywords:** cervical cancer, radiotherapy sensitivity, machine learning algorithms, mitochondrial gene, TMEM38A

## Abstract

**Background:**

Radiotherapy plays a crucial role in the management of Cervical cancer (CC), as the development of resistance by cancer cells to radiotherapeutic interventions is a significant factor contributing to treatment failure in patients. However, the specific mechanisms that contribute to this resistance remain unclear. Currently, molecular targeted therapy, including mitochondrial genes, has emerged as a new approach in treating different types of cancers, gaining significant attention as an area of research in addressing the challenge of radiotherapy resistance in cancer.

**Methods:**

The present study employed a rigorous screening methodology within the TCGA database to identify a cohort of patients diagnosed with CC who had received radiotherapy treatment. The control group consisted of individuals who demonstrated disease stability or progression after undergoing radiotherapy. In contrast, the treatment group consisted of patients who experienced complete or partial remission following radiotherapy. Following this, we identified and examined the differentially expressed genes (DEGs) in the two cohorts. Subsequently, we conducted additional analyses to refine the set of excluded DEGs by employing the least absolute shrinkage and selection operator regression and random forest techniques. Additionally, a comprehensive analysis was conducted in order to evaluate the potential correlation between the expression of core genes and the extent of immune cell infiltration in patients diagnosed with CC. The mitochondrial-associated genes were obtained from the MITOCARTA 3.0. Finally, the verification of increased expression of the mitochondrial gene TMEM38A in individuals with CC exhibiting sensitivity to radiotherapy was conducted using reverse transcription quantitative polymerase chain reaction and immunohistochemistry assays.

**Results:**

This process ultimately led to the identification of 7 crucial genes, viz., GJA3, TMEM38A, ID4, CDHR1, SLC10A4, KCNG1, and HMGCS2, which were strongly associated with radiotherapy sensitivity. The enrichment analysis has unveiled a significant association between these 7 crucial genes and prominent signaling pathways, such as the p53 signaling pathway, KRAS signaling pathway, and PI3K/AKT/MTOR pathway. By utilizing these 7 core genes, an unsupervised clustering analysis was conducted on patients with CC, resulting in the categorization of patients into three distinct molecular subtypes. In addition, a predictive model for the sensitivity of CC radiotherapy was developed using a neural network approach, utilizing the expression levels of these 7 core genes. Moreover, the CellMiner database was utilized to predict drugs that are closely linked to these 7 core genes, which could potentially act as crucial agents in overcoming radiotherapy resistance in CC.

**Conclusion:**

To summarize, the genes GJA3, TMEM38A, ID4, CDHR1, SLC10A4, KCNG1, and HMGCS2 were found to be closely correlated with the sensitivity of CC to radiotherapy. Notably, TMEM38A, a mitochondrial gene, exhibited the highest degree of correlation, indicating its potential as a crucial biomarker for the modulation of radiotherapy sensitivity in CC.

## 1 Introduction

Cervical cancer (CC) continues to be a significant health concern for the global female reproductive system, with the highest incidence and mortality rates observed in countries with the lowest Human Development Index (1, 2). Sustained infection with high-risk human papillomavirus (HPV) 16 and 18 accounts for 70% of CC cases (3). The primary treatment modalities for CC encompass surgical intervention, chemotherapy, and radiotherapy, with radiotherapy playing a pivotal role in its therapeutic strategy (4). Approximately half of cancer patients will undergo radiation therapy as a component of their treatment protocol (5). However, due to the current state of radiotherapy, its effectiveness is restricted to a specific subset of patients, and the significant adverse effects it produces negatively impact on the quality of life of patients. It is worth noting that the resistance of cancer cells to radiotherapy continues to be a prominent factor contributing to treatment failure. However, the precise mechanisms responsible for this resistance are not yet fully understood (6–8). Thus, the investigation and modulation of essential radiation-sensitive targets emerge as a paramount undertaking to enhance the effectiveness of radiotherapy.

Mitochondria is the most important “energy factory” of cells and the energy source for almost all cellular functions (9). Mitochondria participate in cellular energy metabolism, cell differentiation, apoptosis, oxidative stress, calcium homeostasis regulation, etc., and play a crucial role in maintaining normal physiological processes of cells (10, 11). It will cause disease when mitochondrial structure and function are abnormal. The study found that during the process from Intraepithelial neoplasia to cervical cancer, the mtDNA content gradually increases (12). Some scholars believe that melatonin can enhance sensitivity of cervical cancer to cisplatin therapies through increase mitochondrial cell apoptosis (13). Previous studies have shown that mitochondria-targeting treatment strategy enhance the radiotherapy sensitivity (14). Au25(SG)18 clusters can regulate ROS accumulation in mitochondria to achieve a good therapeutic effect (15). Recent studies have shown that HMGCS1 improves the efficacy of radiotherapy for cervical cancer by controlling mitochondrial gene expression (16). Therefore, it is necessary to explore the specific mechanism and immune significance of mitochondrial genes in cervical cancer radiotherapy tolerance.

The present study entails screening differentially expressed genes (DEGs) and their association with the sensitivity of radiotherapy in CC to explore the specific mechanisms through which crucial genes facilitate the infiltration of immune cells. Differential and functional correlation analyses were conducted utilizing the TCGA database. Furthermore, a set of 7 core genes was identified using machine learning algorithms. Subsequently, an analysis was conducted to determine their association with immune cells and pathways. Following this, a risk model was constructed and a comprehensive analysis of the drug was conducted. Experimental validation demonstrated an increased expression of the mitochondrial gene TMEM38A in patients with CC who were responsive to radiotherapy. This finding, thus, provides a novel theoretical framework for comprehending the regulatory mechanisms underlying the effectiveness of radiotherapy in treating CC.

## 2 Materials and methods

### 2.1 Transcriptome data collection and sample grouping

To determine the genes that are associated with sensitivity to radiotherapy in CC, a retrospective collection of data was conducted using the TCGA database, inclusion criteria: Provide RNA-Seq data set of cervical cancer patients, Contains tissues from insensitive patients and sensitive patients after cervical cancer radiotherapy. comprising 126 patients who received radiotherapy were divided into “RT-sensitive” group and “RT-resistant” group based on different outcomes after treatment. Individuals demonstrating “disease stability” and “disease progression” were classified as part of RT-resistant, while those exhibiting “complete remission” and “partial remission” were assigned to RT-sensitive. The preprocessing process of microarray data is to remove empty probes and probes corresponding to multiple genes at the same time. When multiple probes correspond to the same gene, the average expression value of these probes is used as the expression value of the gene. In EdgeR, in-sample normalization is performed by default using the TMM algorithm. Following a thorough and stringent selection process, a total of 10 samples from the control group and 116 samples from the treatment group were finally selected to be included in the study.

### 2.2 Identification of DEGs

As mentioned above, DEGs between the control group and treatment group samples were meticulously screened. Specifically, DEGs were identified using the “Limma” R package, and genes satisfying the stringent criteria of an adjusted p-value <0.05 and an absolute log2 fold change (|log2Fc|) ≥1 were deemed statistically significant.

### 2.3 Functional enrichment analysis of DEGs associated with radiotherapy sensitivity

Enrichment analyses were performed using Gene Ontology (GO) terms and the Kyoto Encyclopedia of Genes and Genomes (KEGG) to elucidate the signaling pathways involved in the identified radiotherapy sensitivity-related DEGs. The analysis was carried out in detail as follows: The “ClusterProfiler” R package was employed to perform functional enrichment analysis of the DEGs. Based on a statistical threshold criterion with an adjusted p-value <0.05, significantly enriched GO and KEGG pathways were identified among DEGs.

### 2.4 Selection of key genes using machine learning algorithms

Expanding on the previously identified DEGs, we used the least absolute shrinkage and selection operator (LASSO) as a compressive estimation method and selected “glmnet” package in R. Furthermore, the value of the penalty parameter (λ) filters out the 10-fold cross-validation with the lowest deviation anomaly probability. After regression analysis, only genes with non-zero coefficients were retained. Random forest (RF) classification was used to evaluate the predictive accuracy of data with a relative importance greater than 0.5. RF classification was implemented using the “randomForest” package in R. After LASSO and RF analysis, the repeated genes were used as central genes. Finally, the results were visualized by constructing a Venn diagram using the “venneuler” R package.

### 2.5 Immune infiltration analysis

The annex of the reference (9) yielded gene sets for 16 immune cells and 13 immune-related pathways. Using these 29 gene sets and gene expression matrices as input files, single sample gene set enrichment analysis (ssGSEA) was conducted using “gsva” R on all samples. Subsequently, the infiltration scores for 16 immune cells and the activity of 13 immune-related pathways were calculated for all samples.

### 2.6 Unsupervised clustering of core genes

Initially, 7 core genes were identified using the methods described above. The patients were categorized into distinct molecular subtypes based on the expression patterns of these key genes employing R packages. We configured the clustering with a maximum K value of 9, identified the most relevant clusters via consistency scoring, and validated the clustering using principal component analysis (PCA). Subsequently, we utilized the Limma and pheatmap R packages to examine the differential gene expression of the core genes between samples from distinct clusters.

### 2.7 Development of diagnostic features and risk model for CC’s radiotherapy sensitivity

An artificial neural network (ANN) was used to construct a diagnostic model based on the core genes associated with CC’s sensitivity to radiotherapy. The performance of the diagnostic model was evaluated using receiver operating characteristic (ROC) analysis. To summarize, a nomogram was constructed using the expression levels of core genes to predict the radiotherapy sensitivity of patients with CC, and ROC curves were utilized to evaluate the efficacy of this model.

### 2.8 Prediction of drug sensitivity for core genes

CellMiner (http://discover.nci.nih.gov/cellminer/) predicted the association between key genes expression and drug sensitivity. Data are displayed using the “impute”, “limma” and “ggpubr” packages in R.

### 2.9 Functional enrichment analysis of core genes using ssGSEA

Single-sample gene enrichment analysis (ssGSEA) was used to solve the specific mechanism of action of key genes on the effects of radiotherapy in cervical cancer. The limma package, org.Hs.eg.db, and the clusterProfiler package performed the analysis and visualization of diagnostic genes for ssGSEA.

### 2.10 RT-qPCR validation

6 samples were collected from The Affiliated Hospital of Youjiang Medical University for Nationalities, including 3 cases in the “RT-sensitive” group and 3 cases in the “RT-resistant” group. Serum samples from radiotherapy patients with CC were collected for reverse transcription polymerase chain reaction (RT-PCR) validation to validate the results of the predictive analysis. The ethical approval for this protocol was obtained from the Ethics Committee of the Affiliated Hospital of Youjiang Medical University for Nationalities. These samples store in a 4°C refrigerator immediately after collection. The total RNA was extracted using the TRIZOL reagent in accordance with the reagent kit instructions. DNA purity and concentration were determined using a Nanodrop spectrophotometer. If OD260/280 is between 1.8 and 2.0 and OD260/230≥2.0, the purity and integrity of the RNA are sufficient for further experiments. Following the reverse transcription of RNA into cDNA, a fluorescence-based quantitative PCR reaction was carried out. These steps are performed on ice and samples are stored at -20°C. The reaction conditions were as follows: 95°C for 10 min, then 40 cycles of 95°C for 2s, 60°C for 20s, and 70°C for 10s. Glyceraldehyde 3-phosphate dehydrogenase (GAPDH) served as the internal standard, and the results were quantified utilizing the 2^-ΔΔCt^ method, with primer information were listed as follows: GAPDH-F: 5′-CAGGAGGCATTGCTGATGAT-3′; GAPDH-R: 5′-GAAGGCTGGGGCTCATTT-3′; TMEM38A-F: 5′-TGTGTCTGCTTCCTGCCTGTG-3′; TMEM38A-R: 5′-CCGTGGTGGTAGTGGTGATGG-3′.

### 2.11 Immunohistochemistry

Paraffin sections of tumor tissues were collected from patients of each group, and immunohistochemical staining was performed, as per the guidelines provided. Paraffin sections were first deparaffinized in xylene for 10 minutes, then dehydrated in graded alcohols and rinsed with distilled water. Sections were then incubated with goat serum for 30 min at 37°C to prevent nonspecific staining. Next, primary antibody, TMEM38A (1:100, Sino Biological), incubation was conducted overnight at 4°C. Wash three times with PBS, 5 minutes each time, add rabbit secondary antibody, and incubate at 37°C for 90 minutes. Diaminobenzidine (DAB) chromogenic kit was used for chromogenic development, followed by hematoxylin counterstaining and separation of the chromogenic product with hydrochloric acid alcohol. The sections were cleared in xylene for 5 minutes, coated with neutral glue. The positive expression rate of TMEM38A was determined by observing it under a light microscope, and five distinct fields were randomly chosen for the calculation. Positive expression rate = positive cells/total number of cells ×100%. Three independent experiments were repeated.

### 2.12 Statistical analysis

R 4.0.5 statistical software was used to complete the analysis. We used the t-test and Wilcoxon rank-sum test for quantifying variables. Quantitative data: When the variances are homogeneous, use the unpaired t test to compare the two groups; when the variances are not homogeneous, use the Wilcoxon rank-sum test. As the amount of data lost is small, this shouldn’t be a big problem. Methods for deleting missing data are also optional. If outliers are found, they are replaced with the mean. Furthermore, Spearman correlation analysis was used to investigate the correlation between DEGs and immune cell infiltration. *P* < 0.05 was considered statistically significant.

## 3 Results

### 3.1 Identification of DEGs and enrichment analysis

**Figure 1:**
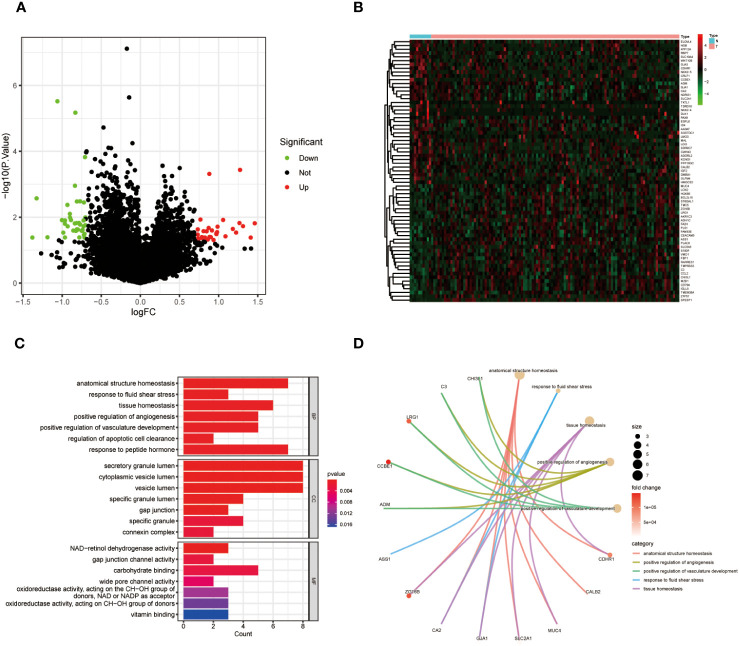
**(A)** Volcano map of differentially expressed genes in CC; **(B)** A heat map of differentially expressed genes in CC. **(C)** GO enrichment analysis of DEGs in CC. **(D)** KEGG pathway analyses of DEGs in CC.

TCGA database analysis revealed a total of 69 DEGs between “RT-sensitive” group and “RT-resistant” group, 31 of which were upregulated and 38 of which were downregulated. **Figures 1A**, **B** depicts the volcano plot and heatmap illustrating DEGs. GO and KEGG enrichment analyses showed that DEGs were mainly enriched in “vasculature development”, “apoptotic cell clearance” and “connexin complex” (**Figures 1C, D**). The ssGSEA results suggest that key genes are closely associated with pathways such as Cell cycle, DNA replication, and p53 signaling pathway (**Supplementary Figure 1**).

### 3.2 Machine learning-based identification of radiotherapy biomarkers in CC

**Figure 2:**
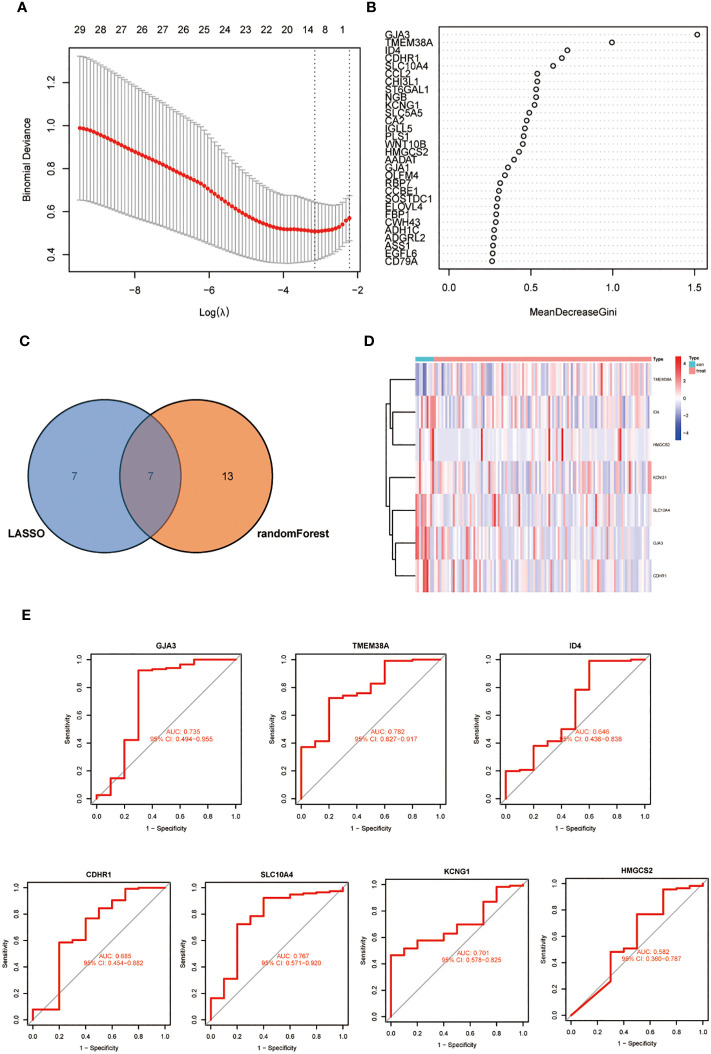
**(A)** LASSO coefficient profiles of the 14 genes that met the prognostic criteria initially. **(B)** RandomForest error rate versus the number of classification trees. **(C)** Venn diagram of genes extracted from LASSO and RF methods. **(D)** A heat map of differentially expressed 7 genes in CC. **(E)** The diagnostic value of the 7 critical genes was studied using ROC assays.

We further refined our selection from the initial set of 69 DEGs using machine learning techniques. LASSO regression uses the compressed estimation method to construct a penalty function based on the least squares method, compressing the regression coefficients while shrinking some regression coefficients to zero. It is currently one of the main methods for processing multicollinear data. we identified 14 feature genes (**Figure 2A**). The RF algorithm simultaneously generated 20 feature genes. Through the intersection of genes derived from both methods, we identified 7 feature biomarker genes, including GJA3, TMEM38A, ID4, CDHR1, SLC10A4, KCNG1 and HMGCS2 (**Figure 2B**). Subsequently, a Venn diagram was generated to represent this common subset visually (**Figure 2C**). **Figure 2D** is a graphical representation of the expression levels of these seven essential genes in samples from radiotherapy-sensitive and radiotherapy-resistant CC patients. The AUC values of all 7 diagnostic genes (**Figure 2E**).

### 3.3 Immunoinfiltration analysis

**Figure 3:**
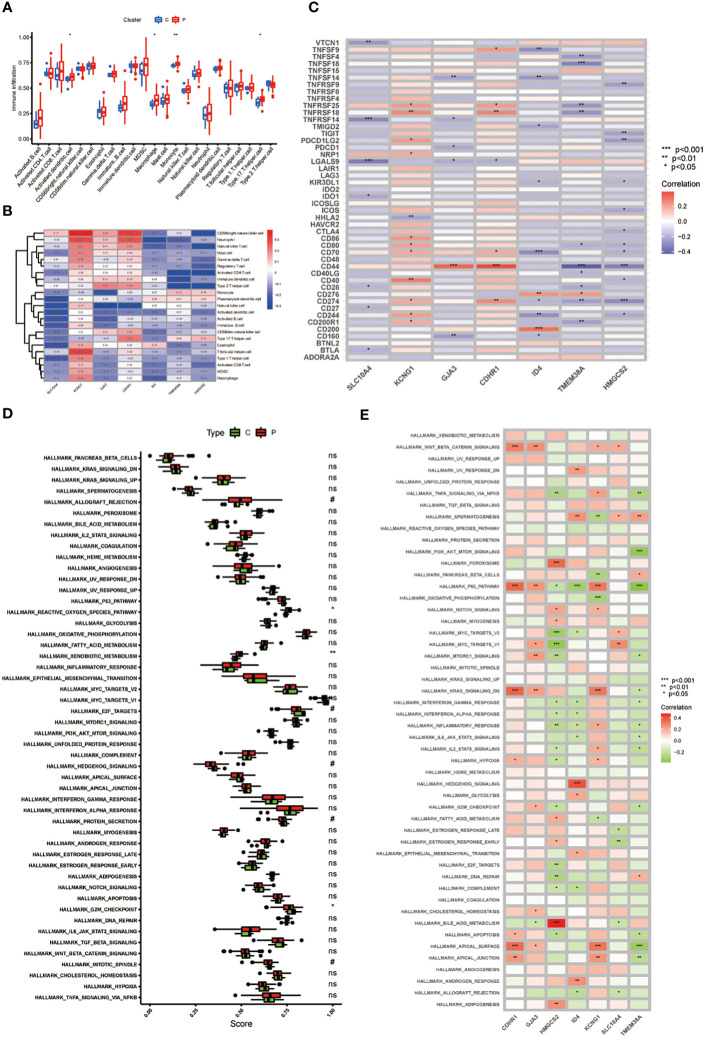
**(A)** The expression of immune cells in CC; **(B)** 7 genes with immune-related functions; **(C)** Immune checkpoint expression in 7 key genes; **(D)** Enrichment analysis in the CC; **(E)** The heat map of GJA3, TMEM38A, ID4, CDHR1, SLC10A4, KCNG1, and HMGCS2 correlation with pathway. P values (*P < 0.05; **P < 0.01; ***P < 0.001). ns, not statistically significant.

As depicted in **Figure 3A**, Our findings revealed that key genes related to immune cells such as Natural killer cell, Neutrophil, CD4 T cells and Type 2 T helper cell, all with p <0.01 (**Figure 3B**). Simultaneously, the expression of these 7 core genes exhibited significant correlations with the expression of numerous immune checkpoints, including CD44, TNFSF14, TNFRSF18 and so on (**Figure 3C**). Finally, the pathways were analyzed and the results suggest that the key genes are closely related to pathways such as reactive oxygen species pathway, xenobiotic metabolism, E2F targets, G2M checkpoint and mitotic spindle. Notably, the functional enrichment analysis results revealed a negative correlation between TMEM38A and the PI3K/AKT/MTOR pathway (**Figures 3D, E**).

### 3.4 Unsupervised clustering of core genes and construction of the risk model

**Figure 4:**
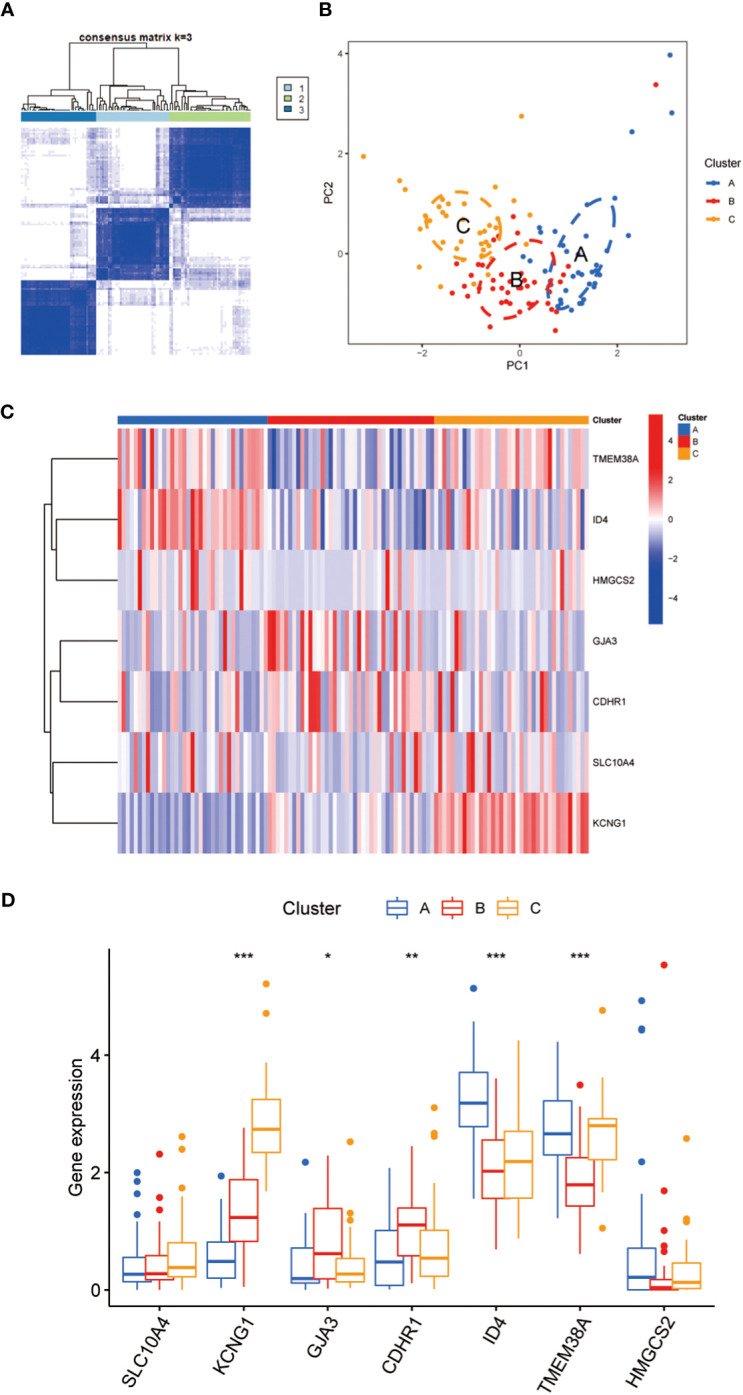
**(A)** To defined three clusters and the consensus matrix heat map of their related regions. **(B)** PCA analysis showed significant transcriptome differences between the three subtypes. A is shown in blue, B in red, C in orange, and there is no sample overlap between them. **(C)** The hot map showed that 7 key genes were significantly different between class A, B and C. **(D)** The Box diagram showed that 7 key genes were significantly different between class A, B and C Asterisks indicate statistical P values (*P<0.05; **P<0.01; ***P<0.001).

**Figure 5:**
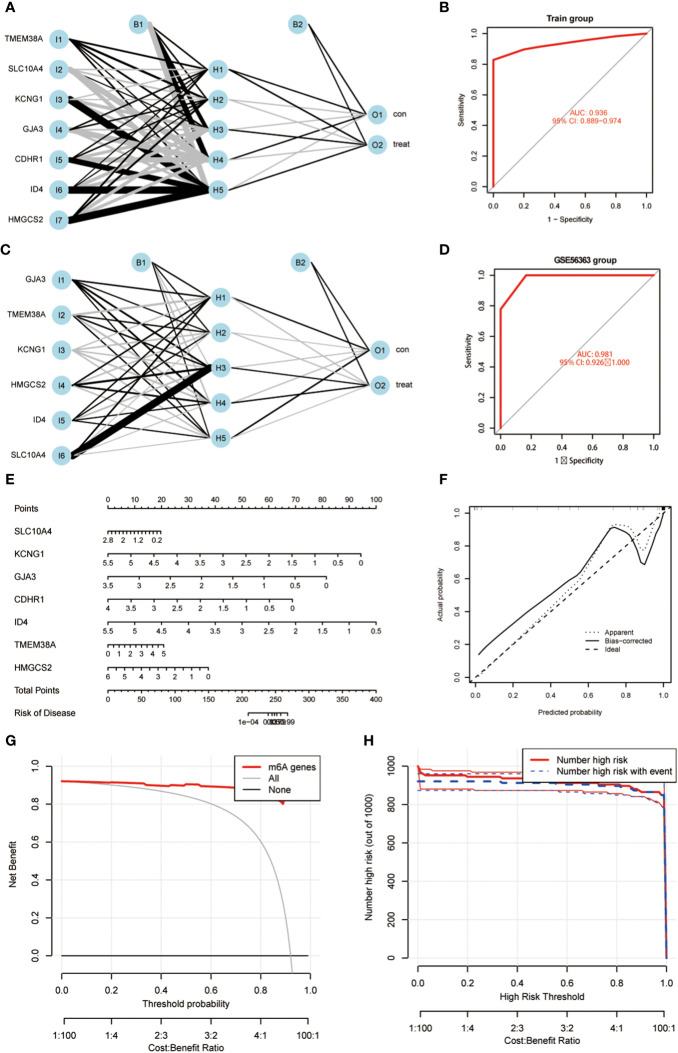
**(A)** An artificial neural network (ANN) was used to construct a diagnostic model; **(B)** ROC curve analysis was performed to validate the model; **(C)** ANN was used to construct a GEO validation dataset of GEO56363; **(D)** ROC curve analysis was performed to validate the model. **(E)** A nomogram was constructed to predict the probability in CC progress. **(F–H)** Validation of the predictive ability of the nomogram model.

Using the 7 core genes described previously, we performed unsupervised clustering analysis on patients with CC, classifying CC samples into 3 distinct molecular subtypes: Cluster A, Cluster B, and Cluster C (**Figure 4A, B**). Comparative analysis among the three clusters revealed significant differences in gene expression. In particular, the expression levels of KCNG1 and TMEM38A were significantly higher in Cluster C than in Clusters A and B. In contrast, GJA3 and CDHR1 demonstrated significantly higher expression in Cluster B than in Clusters A and C. Additionally, ID4 exhibited higher expression in Cluster A than in Clusters B and C (**Figures 4C, D**). Meanwhile, we developed a diagnostic model for the radiotherapy sensitivity of CC within the TCGA dataset (**Figures 5A, C**). ROC curve analysis was then performed to validate the model, yielding an AUC value of 0.936 (**Figures 5B, D**). In predicting the radiotherapy sensitivity of CC, the diagnostic model described above demonstrates strong discriminative abilities. Accordingly, it has the potential for application in clinical settings, aiding in evaluating radiotherapy tolerance in patients with CC (**Figure 5E**). In addition, for user-friendliness, we have successfully developed a nomogram for assessing the risk of radiotherapy tolerance for CC based on the 7 core genes mentioned above, and verified the accuracy of the model (**Figures 5F–H**).

### 3.5 Selection of candidate drugs and validation of TMEM38A

**Figure 6:**
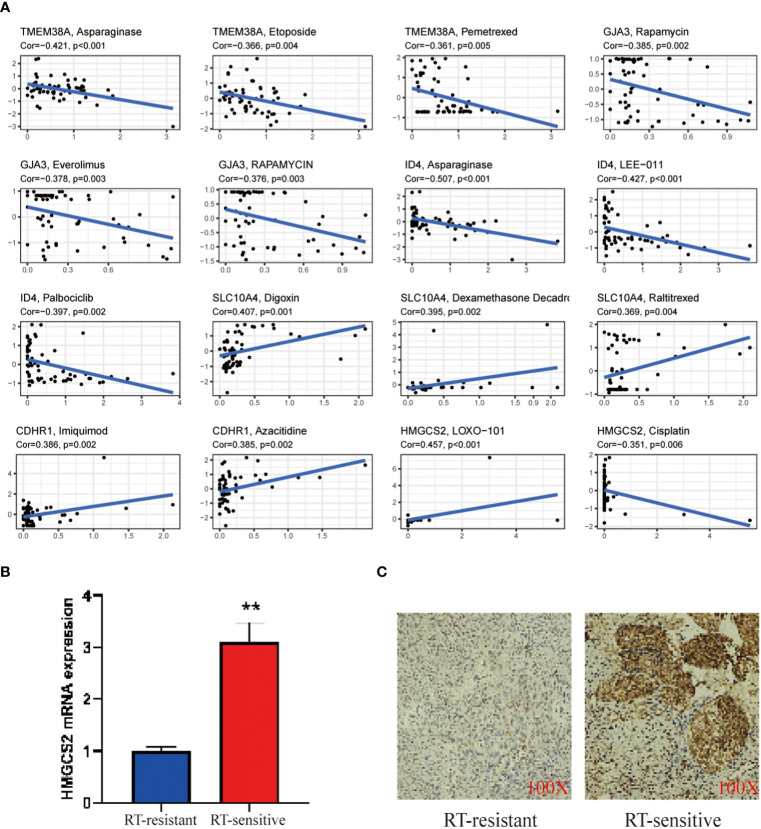
**(A)** Correlation analysis of TMEM38A, GJA3, ID4, SLC10A4, CDHR1 and HMGCS2 corresponding to drug sensitivity in the CellMiner database; **(B)** RT-PCR validation of the mitochondrial gene TMEM38A between treat and control; **(C)** IHC validation of the hub gene TMEM38A between treat and control. The treat group was radiotherapy-sensitive patients and the control group was radiotherapy-resistant patients. as mean ± SEM (**p < 0.01.).

In order to enhance the sensitivity of radiotherapy in CC, we conducted a focused drug analysis utilizing the 7 core genes that are linked to radiotherapy sensitivity. Moreover, within clinical environments, TMEM38A was found to have a negative correlation with the drugs etoposide and pemetrexed. Meanwhile, ID4, GJA3, SLC10A4,CDHR1 and HMGCS2 correlated with the drugs azacitidine, everolimus, rapamycin, raltitrexed, imiquimod, LOXO-101 and cisplatin (**Figure 6A**). Validation through RT-qPCR and immunohistochemistry assays revealed that the mitochondrial gene TMEM38A showed lower expression levels in CC tissues that exhibited resistance to radiation therapy in comparison to tissues that displayed sensitivity to radiation therapy (**Figure 6B**). These findings confirm that the increased expression of TMEM38A in radiation therapy-sensitive CC tissues is consistent with the findings observed in the TCGA database (**Figure 6C**).

## 4 Discussion

Currently, the majority of cancer patients require combined treatments, consisting of radiation therapy alone or in conjunction with other therapeutic methods (17–20). Among these, the combination of surgical procedures and radiotherapy is a commonly utilized clinical approach for treating CC (21). Radiation therapy can potentially increase survival rates, enhance quality of life, and reduce adverse effects in patients with CC (22). Nonetheless, numerous clinical studies have uncovered variations in patients’ innate sensitivity to radiation, particularly among those with early or advanced cervical cancer. This variation can cause some individuals to develop resistance to radiation therapy (7, 8). Consequently, a subset of patients with CC undergoing radiation therapy may fail to achieve effective local disease control, significantly impacting treatment efficacy and long-term prognosis (23, 24). Current prognostic and risk assessment systems for CC do not incorporate an evaluation of radiotherapy sensitivity. Consequently, the quest to identify biological biomarkers or therapeutic targets that correlate with radiotherapy sensitivity in patients with CC is of great importance.

In recent years, mitochondria have assumed a central role in the genesis and progression of cancer. The process of mitochondrial oxidative phosphorylation results in the release of ROS, causing mitochondrial dysfunction. These damaged mitochondria release apoptosis-related proteins, such as cytochrome C and caspases, which ultimately induce cellular apoptosis. Studies have indicated that the inhibition of mitochondrial fragmentation effectively inhibits radiation-induced cellular apoptosis, thereby enhancing the radiotherapy resistance of CC cells (25). Simultaneously, tumor cells experience a substantial increase in mitochondrial metabolic activity and ROS production, which can activate the p53 pathway and induce autophagy. It has been observed that the radiation-induced induction of autophagy in cells increases the radiosensitivity of CC cells. Nonetheless, the precise role and underlying mechanisms of mitochondrial autophagy in the context of radiotherapy resistance in CC remain unknown. Due to the inherent resistance of CC cells to radiation, patients undergoing radiotherapy are frequently at a higher risk for disease recurrence. Consequently, there is an urgent need to identify radiosensitive biomarkers to augment the radiation response in CC, ultimately increasing patient survival rates. The silencing of the mitochondrial uncoupling protein UCP2 increases the sensitivity of HeLa cells to radiation-induced DNA damage. This silencing simultaneously induces increased apoptosis, G2/M phase cell cycle arrest, increased mitochondrial ROS levels, and radiation sensitization in HeLa cells (26).

First of all, the present study investigated potential DEGs associated with radiosensitivity in CC by Using bioinformatics analysis. Subsequently, using LASSO regression and RF analysis, a subset of seven key genes, namely GJA3, TMEM38A, ID4, CDHR1, SLC10A4, KCNG1, and HMGCS2, was refined to represent the intersection of the identified DEGs. Earlier studies suggest that the gap junction alpha-3 protein (GJA3) may facilitate the intracellular exchange of ions, small molecules, and fluids (27). GJA3 is the gene responsible for encoding the gap junction protein Cx46, which facilitates the transfer of messenger molecules between neighboring cells (28–30). Moreover, research has demonstrated that autophagy can enhance the radiosensitivity of non-small cell lung cancer (31, 32). Consequently, it is conceivable that GJA3 could modulate the radiosensitivity of CC by regulating the process of autophagy. Transmembrane Protein 38A (TMEM38A), also known as trimeric intracellular cation channel type A (TRIC-A) (33), functions as a monovalent cation channel essential for facilitating rapid intracellular calcium release. TRIC-A plays a crucial role in modulating calcium ion transport within cardiac muscle cells (34, 35), and its expression is observed during the early stages of zebrafish development (34). ID4 is intricately associated with cancer, with processes such as proliferation, differentiation, migration, and invasion involving numerous cancer types (36–39). Cadherin-related family member 1 (CDHR1), a calcium-dependent cell-cell adhesion membrane protein, has prognostic significance in glioblastoma (GBM), with decreased CDHR1 expression being associated with poor clinical outcomes (40, 41). SLC10A4 is a vesicular monoamine and cholinergic transporter protein that plays an essential role in regulating dopamine homeostasis and neuroregulation within the body (42). It also serves as a regulator of extracellular degranulation and mast cell-related intracellular responses (43). In contrast, KCNG1, a gene involved in fatty acid metabolism (FAM), has a strong correlation with the prognosis of head and neck squamous cell carcinoma (31). HMGCS2, also known as 3-hydroxy-3-methylglutaryl-coenzyme A synthase 2, is a key ketogenic enzyme responsible for facilitating ketone production and regulating tumor proliferation and metastasis (26, 44). The close association of these genes with radiosensitivity in CC and their underlying mechanisms, calls for additional in-depth research and investigation. Interestingly, the mitochondrial gene TMEM38A is also associated with the immune checkpoints CD44 and TNFSF18, so the immune checkpoint inhibitor PD-1 may be an immunotherapeutic option for targeting TMEM38A.

In the next part, as everyone knows, immune infiltration plays a crucial role in cancer radiotherapy. The purpose of this study was also to identify potential central genes associated with radiotherapy for CC and immune infiltration. The results revealed a significant upregulation of immune cells, such as NK cells, in the CC tissues with the most notable differences. NK cells, a subset of natural lymphoid cells, exhibit cytotoxic activity against tumor cells and suppress tumor metastasis by inhibiting cancer cell proliferation, migration, and distant organ colonization (45). The importance of NK cells to the efficacy of radiotherapy has been demonstrated in earlier studies. Nonetheless, the toxic side effects of radiotherapy can impair the normal immune function of the host’s NK cells (46). Through analysis, it has been determined that radiosensitive patients actively participate in pathways involving ROS, exogenous compound metabolism, and the cell cycle. Furthermore, the identified key genes are primarily related to NK cells. It is worth noting that previous research has validated NK cells as the most promising target for enhancing the immune response in patients with CC, making them highly responsive to radiotherapy (47). The GJA3, TMEM38A, CDHR1, and HMGCS2 genes closely correlate with the immune checkpoint CD44, whereas TMEM38A, a mitochondrial gene, demonstrates a robust association with TNFSF18. These observations may provide new insights into the role of mitochondria in immunotherapy for CC. Despite the extensive research on tumor metabolism, mitochondria have received limited attention as potential contributors to prognostic models in cancer. However, they hold significant promise as emerging biomarkers for CC. This possibility could shed light on new targets for CC immunotherapy and provide novel potential biomarkers and therapeutic strategies for CC.

In the end, CC comprises subtypes such as invasive squamous cell carcinoma, adenocarcinoma, adenosquamous carcinoma, adenoid basal carcinoma, villoglandular adenocarcinoma, and endometrioid carcinoma. A growing body of evidence highlights the significance of subtype classification in the diagnosis, treatment, and prognosis of CC. Furthermore, earlier studies have indicated that patients with cervical squamous cell carcinoma exhibit poorer radiotherapy outcomes compared to those with cervical adenocarcinoma. Consequently, CC subtyping may serve as a more accurate guide for treatment decisions (16, 48). Herein, the unsupervised clustering analysis revealed the presence of three distinct subtypes. Accordingly, KCNG1 and TMEM38A exhibited significantly higher expression levels in subtype C compared to subtypes A and B. In contrast, GJA3 and CDHR1 exhibited significantly higher expression in subtype B than in subtypes A and C. Additionally, ID4 expression was higher in subtype A than in subtypes B and C. Concurrently, RT-qPCR and IHC experiments were conducted to confirm the expression levels of TMEM38A in radiotherapy patients. The results revealed that TMEM38A expression was low in radiotherapy-resistant patients but high in radiotherapy-sensitive patients with CC. In addition, immunohistochemistry validation supported the elevated expression of TMEM38A in radiotherapy-sensitive patients. These findings hold promise for the development of novel therapeutic avenues and individualized treatment strategies for CC radiotherapy.

In general, the expression of DEGs and their prognostic significance in CC radiotherapy were systematically analyzed in the present study, Accordingly, TMEM38A was identified as one of seven core genes due to its association with mitochondrial function. In addition, earlier studies suggest that tumor angiogenesis serves as an independent prognostic factor for disease-free survival in patients with CC, and anti-angiogenic therapies have been demonstrated to be effective in managing late-stage or recurrent CC cases (31, 49, 50). we performed separate analyses to determine the relationship between key genes involved in radiotherapy for CC and immune cells. Accordingly, a close association was identified between these genes and tumor-infiltrating immune cells, including natural killer (NK) cells, neutrophils, and CD4+ T cells in patients with CC. Furthermore, significant correlations were observed between the mitochondrial gene TMEM38A and the immune checkpoints CD44 and TNFSF18. Enrichment analysis of both GO and KEGG signaling pathways indicates that the aforementioned DEGs regulate angiogenesis and vascular development. In addition, the mitochondrial gene TMEM38A is negatively related to the PI3K/AKT/MTOR pathway. Some cancers have encoded mitochondrial tricarboxylic acid cycle (TCA) enzyme that produces oncogenic metabolites. Removal of mtDNA inhibits tumorigenesis. Therefore, TMEM38A may modulate the efficacy of radiotherapy by affecting angiogenesis in cervical cancer through the PI3K pathway and the immune system. These findings may offer novel insights into the role of mitochondria in immunotherapy for CC, as well as potential new therapeutic biomarkers and treatment strategies for CC. and anti-angiogenic therapies have been demonstrated to be effective in managing late-stage or recurrent CC cases.

The study also has some limitations that deserve discussion. Firstly, each subtype system has different interpretations of the molecular classification of colorectal cancer due to differences in patient cohorts, sequencing platforms, bioinformatics analysis methods, and data analysis across studies. Although we have identified a correlation between 7 genes and radiotherapy sensitivity, their specific mechanisms need to be further verified the roles of identified genes and pathways through a large amount of clinical data, cell experiments, and animal experiments. At the same time, this study also suffers from relatively small amount of data with missing values, and more experiments and data sets are needed for validation. Next, we will further verify the impact of key genes on cervical cancer chemotherapy through experiments such as CCK-8, Transwell and wound healing. Finally, we need to experimentally validate key genes and pathways to determine their functions to enable precision radiotherapy technology through targeted therapy. precision radiotherapy can effectively reduce the side effects of radiotherapy for cervical cancer, improve patients’ quality of life, and help achieve the expected effects of radiotherapy.

## 5 Conclusions

The findings of the present study demonstrate that the genes GJA3, TMEM38A, ID4, CDHR1, SLC10A4, KCNG1, and HMGCS2 exhibit a notable correlation with radiotherapy sensitivity in CC, with TMEM38A exhibiting the strongest association. Accordingly, the identified biomarker demonstrates potential as a significant factor in the modulation of radiotherapy sensitivity in CC.

## Data availability statement

The datasets presented in this study can be found in online repositories. The names of the repository/repositories and accession number(s) can be found below: https://www.ebi.ac.uk/pdbe/emdb/, Microarray data for human CC and control specimens were obtained from the TCGA database (https://www.cancer.gov/CESCg/research/genome-sequencing/tcga).

## Ethics statement

The studies involving humans were approved by the Institutional Research Ethics Committees of the Affiliated Hospital of Youjiang Medical University for Nationalities (YYFY-LL-2023-136). The studies were conducted in accordance with the local legislation and institutional requirements. The participants provided their written informed consent to participate in this study.

## Author contributions

JW: Data curation, Formal Analysis, Writing – original draft. XM: Formal analysis, Writing – review & editing. HL: Formal analysis, Writing – review & editing. HJ: Supervision, Writing – review & editing. YX: Supervision, Writing – review & editing. XW: Supervision, Writing – review & editing. ZH: Visualization, Writing – review & editing. ST: Visualization, Writing – review & editing. HC: Visualization, Writing – review & editing. MD: Conceptualization, Formal analysis, Writing – original draft. YL: Conceptualization, Writing – review & editing. GS: Conceptualization, Funding acquisition, Writing – review & editing.
